# Business Models of eHealth Interventions to Support Informal Caregivers of People With Dementia in the Netherlands: Analysis of Case Studies

**DOI:** 10.2196/24724

**Published:** 2021-06-03

**Authors:** Hannah Liane Christie, Lizzy Mitzy Maria Boots, Ivo Hermans, Mark Govers, Huibert Johannes Tange, Frans Rochus Josef Verhey, Majolein de Vugt

**Affiliations:** 1 Alzheimer Centrum Limburg Maastricht University Maastricht Netherlands; 2 Betawerk Heerlen Netherlands; 3 Department of Health Services Research CAPHRI School for Public Health and Primary Care Maastricht University Maastricht Netherlands; 4 Department of Family Practice CAPHRI School for Public Health and Primary Care Maastricht University Maastricht Netherlands

**Keywords:** eHealth, dementia, caregiving, implementation, business modeling

## Abstract

**Background:**

In academic research contexts, eHealth interventions for caregivers of people with dementia have shown ample evidence of effectiveness. However, they are rarely implemented in practice, and much can be learned from their counterparts (commercial, governmental, or other origins) that are already being used in practice.

**Objective:**

This study aims to examine a sample of case studies of eHealth interventions to support informal caregivers of people with dementia that are currently used in the Netherlands; to investigate what strategies are used to ensure the desirability, feasibility, viability, and sustainability of the interventions; and to apply the lessons learned from this practical, commercial implementation perspective to academically developed eHealth interventions for caregivers of people with dementia.

**Methods:**

In step 1, experts (N=483) in the fields of dementia and eHealth were contacted and asked to recommend interventions that met the following criteria: delivered via the internet; suitable for informal caregivers of people with dementia; accessible in the Netherlands, either in Dutch or in English; and used in practice. The contacted experts were academics working on dementia and psychosocial innovations, industry professionals from eHealth software companies, clinicians, patient organizations, and people with dementia and their caregivers. In step 2, contact persons from the suggested eHealth interventions participated in a semistructured telephone interview. The results were analyzed using a multiple case study methodology.

**Results:**

In total, the response rate was 7.5% (36/483), and 21 eHealth interventions for caregivers of people with dementia were recommended. Furthermore, 43% (9/21) of the interventions met all 4 criteria and were included in the sample for the case study analysis. Of these 9 interventions, 4 were found to have developed sustainable business models and 5 were implemented in a more exploratory manner and relied on research grants to varying extents, although some had also developed preliminary business models.

**Conclusions:**

These findings suggest that the desirability, feasibility, and viability of eHealth interventions for caregivers of people with dementia are linked to their integration into larger structures, their ownership and support of content internally, their development of information and communication technology services externally, and their offer of fixed, low pricing. The origin of the case studies was also important, as eHealth interventions that had originated in an academic research context less reliably found their way to sustainable implementation. In addition, careful selection of digital transformation strategies, more intersectoral cooperation, and more funding for implementation and business modeling research are recommended to help future developers bring eHealth interventions for caregivers of people with dementia into practice.

## Introduction

### Background

A recent systematic review [1] showed that very few evidence-based eHealth interventions for informal caregivers of people with dementia have been implemented in practice. eHealth, defined by the World Health Organization as “the use of information and communication technologies (ICT) for health” [2], has the potential to help many people living with physical and mental health issues, including informal caregivers of people with dementia. Informal care constitutes a significant part of dementia care [3,4] and can include helping with household chores, running errands, facilitating social engagement, and coordinating professional care. In the Netherlands, it has been estimated that approximately 10% of the 16 million inhabitants offer some form of informal care [5], whereas an estimated 320,000 informal caregivers provide care specifically for people with dementia [6]. However, informal caregivers have also been shown to experience physical and psychological complaints as a result of this caregiving process [7]. Given the fact that the current worldwide prevalence of dementia (50 million people) is expected to triple by 2050 [8] and the health care systems’ increasing reliance on informal care [9], it is important to support these informal caregivers of people with dementia. Previous research has shown that eHealth interventions to support these caregivers have been effective in improving caregiver outcomes, such as self-efficacy, competence, and knowledge about dementia, and in reducing depressive symptoms [10-18].

In general, eHealth has many potential benefits compared with more traditional face-to-face interventions: it is relatively easy to implement on a larger scale; it has the potential to reach users from various socioeconomic and demographic backgrounds; and it can include extensive personalization, instant delivery, and real-time feedback [19,20]. There is preliminary evidence that web-based tools might be at least as effective as face-to-face interventions in delivering psychiatric support [21], although more research is needed. eHealth as a potential solution to facilitate access to dementia caregiving support is even more crucial in the context of the current COVID-19 pandemic. A recent article in *The Lancet* on the impact of COVID-19 on people with dementia and their caregivers advised care professionals to organize psychoeducation, self-management, and consultations for dementia caregivers on the web [22]. A survey of 1000 Dutch caregivers of people with dementia by Alzheimer Netherlands showed that the COVID-19 pandemic has resulted in the cancelation of day care services for people with dementia as well as insufficient professional and social support. Caregivers mentioned digital contacts, such as video chatting or WhatsApp messaging, as one of the solutions that currently helps them the most [23].

Unfortunately, the implementation of eHealth is often fragmented and short lived and lacks sufficient vision and strategy [24]. In addition to the eHealth interventions being developed in an academic research context, there is also eHealth being developed outside of academia, for instance, by industry and health care organizations [25], from which much can be learned to aid the implementation of eHealth interventions for caregivers of people with dementia originating from the research context. For the most part, these nonacademic interventions are not included in the search strategies used in systematic reviews, as their development and testing are usually not published in academic journals.

**Figure 1:**
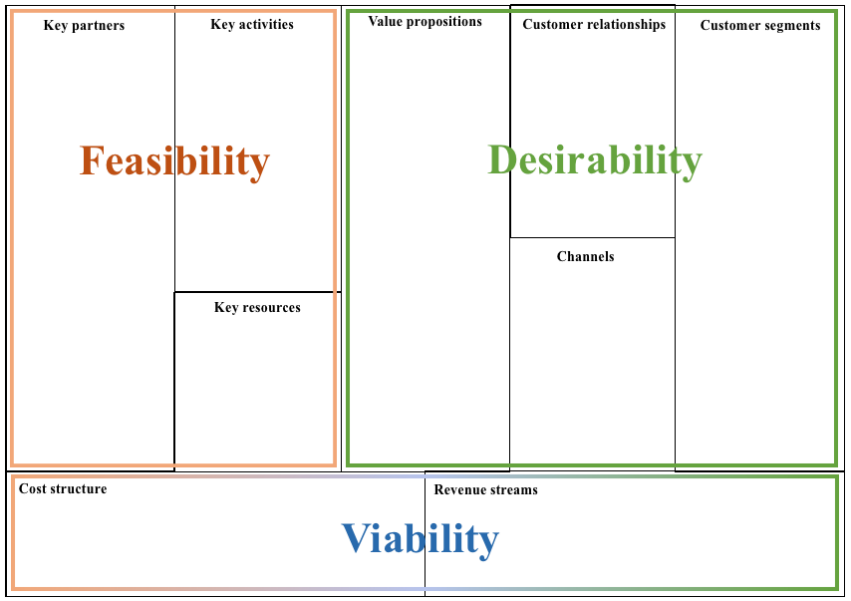
Desirability, feasibility, and viability of the business models.

A useful framework to explore the factors that contribute to the (financial) sustainability of nonacademic interventions is the Business Model Canvas [26]. The Business Model Canvas is an established framework intended to aid in the development and mapping of new and existing business models by demonstrating the product or service’s value proposition, key activities, key resources, key partners, cost structure, customer relationships, distribution channels, and revenue. The Business Model Canvas has previously been used to map business models for eHealth interventions [27-29]. Recently, developers grouped segments of the Business Model Canvas to construct blocks of desirability, feasibility, and viability [30]. Figure 1 (adapted from the study by Osterwalder and Pigneur [26]) illustrates how the 9 elements of the Business Model Canvas can be grouped into these factors.

### Objectives

This study aims to explore how lessons learned from interventions currently being used in practice could aid the implementation of evidence-based interventions for informal caregivers of people with dementia. To accomplish this, this study had 3 aims: (1) to examine a sample of case studies of eHealth interventions to support informal caregivers of people with dementia that are currently used in the Netherlands; (2) to investigate what strategies are used to ensure the desirability, feasibility, viability, and sustainability of the interventions; and (3) to formulate lessons learned to facilitate the implementation of future eHealth interventions.

## Methods

### Study Design

#### Step 1: Identifying eHealth Interventions for Caregivers of People With Dementia

##### Contacting Experts

First, to acquire a sample of eHealth interventions to support caregivers of people with dementia that are being used in practice, experts (N=483) in the fields of eHealth, dementia, and caregiving were contacted via email.

##### Inclusion Criteria

The experts were asked to provide the names of interventions that met the following 4 inclusion criteria: eHealth intervention (1) delivered via the internet; (2) suitable for informal caregivers of people with dementia, (3) available in the Netherlands, either in Dutch or in English; and (4) used in practice. The aim was to receive recommendations for between 5 and 10 interventions that met all criteria, as this would achieve data saturation but still be a small enough number to analyze in depth. The suggested interventions were not meant to serve as an exhaustive overview of all available eHealth interventions for caregivers of people with dementia in the Netherlands. Rather, they are a sample of this type of intervention, which was defined using a systematic approach.

#### Step 2: Qualitative Interviews With eHealth Intervention Providers

After receiving these recommendations, the researchers reached out to the interventions’ contact people with an invitation to participate in a telephone interview about their experiences with their intervention’s implementation. This study used a multiple case study methodology for the analysis. This methodology was chosen for its ability to qualitatively explore complex phenomena within their contexts and explore differences within, as well as between, cases [31]. The focus is on collecting in-depth data from a limited number of cases. To do this, this study made use of semistructured interviews because of their fit with the aim of collecting qualitative, open-ended data, using which it is possible to delve deeply into the thoughts and opinions of the participants. The semistructured interviews consisted of 9 questions. The interview guide can be found in Multimedia Appendix 1. It was constructed by the authors to explore the desirability, feasibility, and viability of the included eHealth cases based on the Business Model Canvas framework [26]. To do this, the interview guide asks the participants questions based on each of the 3 main domains. In addition, data were collected on the description, current use, and business model of the interventions.

### Sample

First, emails with an invitation to provide examples of interventions meeting the 4 inclusion criteria were sent to a diverse sample of experts. Experts (n=330) in the academic field were approached via the INTERDEM mailing list. INTERDEM is an international research network that focuses on psychological care for dementia. Second, 4 emails were sent to experts in the industry (employees of small- and medium-sized enterprises, which usually consist of <250 employees), 10 emails were sent to representatives from dementia and caregiver patient organizations, and 4 emails were sent to clinicians in the field of dementia care (3 psychologists and 1 psychiatrist). Regarding the significant difference in the number of emails sent per subsample, the possibility of approaching researchers via the INTERDEM network (consisting of 330 mailing list contacts) was associated with a much lower response rate, as it was a general mailing instead of a targeted personal mailing. Third, 15 dementia caregivers were asked to provide examples of interventions that met the 4 inclusion criteria during a dementia client panel meeting at Maastricht University. The caregivers were part of the Maastricht University’s dementia client panel. Finally, a notice was placed on the social media channels (Twitter, Facebook, and LinkedIn) of the Nederlandse Vereniging voor Neuropsychologie (NVN; the Dutch Association for Neuropsychology), which was clicked on 120 times across channels, according to NVN.

Table 1 provides an overview of the backgrounds of the included interview participants whose interventions met all 4 criteria and who could be contacted for an interview (the *Results* section provides more details on intervention inclusion).

**Table 1:** Interview participants’ background.

Case study	Intervention name	Background interview participant
1	Partner in Balance	Academia
2	STAR eLearning	Academia
3	OZOverbindzorg	Industry
4	Carenzorgt	Industry
5	(redacted)	Clinic (psychologist)
6	Thinkability	Academia
7	DementieNL (and Myinlife)	Patient organization
8	(redacted)	Clinic (psychologist)
9	Nachtrust bij Dementie	Academia

### Data Collection

First, emails with an invitation to provide examples of interventions that met the 4 inclusion criteria were sent to a diverse sample of experts (N=483). Each of these experts was known to the research team through their own professional networks. Each email was sent once. A total of 15 dementia caregivers were asked face-to-face to provide examples during a dementia client panel meeting at Maastricht University. The notice on the NVN was placed once.

Once interventions were identified, the providers of these interventions were interviewed over the telephone. For the 9 included case studies, interview data were collected using the interview guide in Multimedia Appendix 1. These data were collected between December 2018 and June 2019. In total, 9 interviews were conducted via telephone and transcribed verbatim. On average, the interviews lasted for 25 minutes (SD 11.9). Data were also collected on the description, current use, and business model of the interventions and are represented in an extraction table (refer to the *Results* section).

### Data Analysis

The data were analyzed using a qualitative, multiple case study methodology [32]. HLC conducted the initial analysis using the extraction table and interview transcripts. The first phase of this analysis involved examining the individual cases to understand each intervention, its implementation trajectory, and its business model. Here, the focus was on *explanation building* [33], where the narratives from the interviews were combined with the data from the extraction table to map each sampled intervention in detail.

The second phase involved comparing cases with each other to identify common and differential characteristics. To do this, the interview transcripts were analyzed across cases by assigning codes (desirability, feasibility, and viability) to respondents’ replies that provided more information on these topics. This guided an explanation of which characteristics could contribute to intervention desirability, feasibility, and viability across cases.

In the third phase, the results of this initial analysis were discussed with HLC, LMMB, HJT, MG, and MEDV in a consensus meeting, where differences in interpretation were resolved and external validity was supported. In particular, the authors discussed the value propositions (ie, the elements of the service or product that are intended to make the intervention more attractive to customers) of the included cases; their common characteristics in terms of desirability, feasibility, and viability; and their respective implementation phases and business models. Finally, a member check (where participants were sent the interview transcript for approval) [34] served to support the internal validity of the analysis.

### Ethics

This study did not fall under the scope of the Medical Research Involving Human Subjects Act. Therefore, ethical approval by Maastricht University’s institutional review board was not required. The respondents were verbally informed of the purpose of the study before the interview and gave their consent to the interview being audio recorded and analyzed anonymously. However, it was not possible to guarantee total anonymity because the interventions themselves were discussed by name. Therefore, it was requested that names of interventions not be used for case studies 5 and 8. The participants consented to this, and a *member check* was conducted. Through this member check, all participants (including the participants involved in case studies 5 and 8) approved (in writing) the information in the transcripts being used in this study (with the exception of the names of case studies 5 and 8).

## Results

### Overview

The first section provides an overview of all the interventions suggested by the contacted experts and describes the included case studies. In the second section, the following characteristics of the included case studies are examined: (1) desirability, feasibility, and viability of the interventions; (2) findings on the sustainability of their business models; and (3) lessons learned from the respondents.

### Interventions Suggested by Experts

#### Overview of Interventions

**Figure 2:**
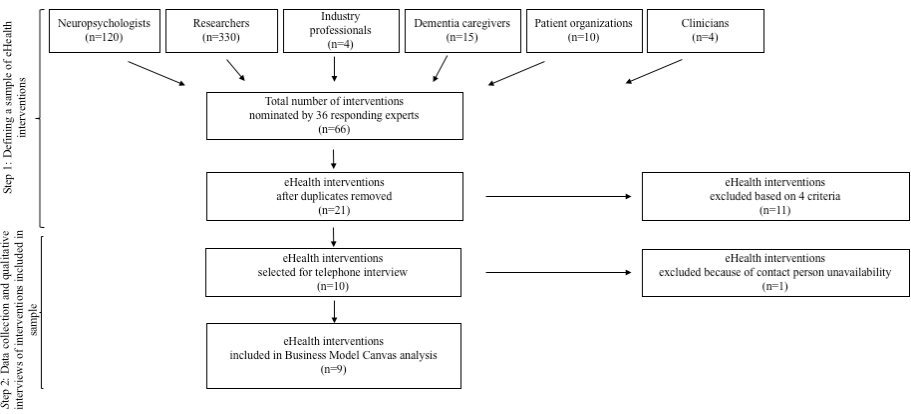
Flowchart of case study inclusion.

A total of 36 responses were received (14 from researchers, 10 from patient organizations, 3 from industry professionals, 4 from clients, and 5 from clinicians), resulting in a response rate of 7.5% (36/483). Although this response rate is rather low, it was deemed well suited for the described purpose of gathering a limited, nonexhaustive sample of cases to be analyzed in depth by multiple case study methodology. A total of 21 interventions were nominated to be part of the sample. Some experts suggested multiple interventions, whereas others did not suggest any interventions. Overall, 11 interventions were excluded based on the 4 criteria described earlier. A final intervention was excluded because nobody could be contacted for the interview, resulting in insufficient data for a case study analysis. Figure 2 depicts a flowchart of the case study inclusion.

#### Included Case Studies

In total, 9 interventions met all 4 criteria and could be contacted for interviews, resulting in their inclusion as case studies. Table 2 provides the names, number of nominations, description, and current use of the included case studies. DementieNL and Myinlife are discussed together, as Myinlife has been integrated into the broader DementieNL platform.

**Table 2:** Extraction table.

Case study	Intervention name	Nominations	Description	Current use (January 2020)	Pricing
1	Partner in Balance	5	Web-based intervention to support caregivers and promote self-management, with coach (a formal caregiver), for informal caregivers (academic origin)	Available on the web (only in participating areas)	€200 (US $243.28) per caregiver per year
2	STAR eLearning	1	Web-based intervention to improve dementia caregiving skills for both formal and informal caregivers (academic origin)	Available on the web (after approval)	€25 (US $30.41) per caregiver per year
3	OZOverbindzorg	1	Web-based platform to connect care partners, for informal caregivers to invite formal caregivers (nonacademic origin)	Available on the web (only in participating regions or municipalities)	Health insurer and municipality jointly pay €1 (US $1.22) per user (calculated over region)
4	Carenzorgt	5	Web-based platform to facilitate the organization of care for people with dementia, for both formal and informal caregivers (nonacademic origin)	Available on the web	Free
5	{redacted}	4	Web-based intervention to provide tips for caring for a person with dementia, with a coach, for both formal and informal caregivers (partial academic origin)	Available on the web	Free if the person with dementia is registered with the care organization (name redacted), €500 (US $608.20) for course if not registered with the care organization (name redacted), and currently not accepting new applicants because of lack of resources
6	Thinkability	1	Web-based cognitive stimulation therapy intervention to improve quality of life, for both people with dementia and informal caregivers (academic origin)	Available	£4.99 (US $7.06) in App Store and Google Play
7	DementieNL (and Myinlife)	7	Web-based platform for dementia care (included caregiver test, web-based dementia training, and “ask an expert”), for informal caregivers. Includes Myinlife, a web-based platform to facilitate the organization of care for people with dementia (partial academic origin)	Available on the web	Free
8	{redacted}	1	Web-based platform that contains both internal and external modules informing about dementia care, for formal and informal caregivers (partial academic origin)	Available on the web	Free if caregiver is a client with Innovate (organization), otherwise €10 (US $12.16)
9	Nachtrust bij Dementie	1	Web-based intervention to inform about night-time unrest in a person with dementia and provide nonpharmaceutical tips, for informal caregivers (academic origin)	Web-based platform, although coaching not currently available because of lack of resources	No pricing model (research project)

### Case Study Characteristics

#### Strategies Relating to Case Study Desirability, Feasibility, and Viability

##### Overview

Table 3 provides an overview of the interview respondents’ answers to the questions about the current desirability, feasibility, and viability characteristics of their interventions. The table synthesizes these responses across case studies and groups them according to the categories of desirability, feasibility, and viability. The characteristics are first presented in the table and described in more detail in the following sections.

**Table 3:** Common case study characteristics, as reported in case study interviews.

Common characteristics		Partner in Balance	STAR eLearning	OZOverbindzorg	Caren-zorgt	(redacted)	Thinkability	DementieNL (and Myinlife)	(redacted)	Nachtrust bij Dementie
**Desirability**										
	Targeted user is mostly caregiver	✓^a^			✓	✓		✓		✓
	Low price (see Table 2 for prices)	✓	✓	✓	✓	✓	✓	✓	✓	✓
	Incorporated into larger systems	✓	✓	✓	✓	✓	✓	✓	✓	
	Up-to-date content	✓	✓	✓		✓	✓	✓	✓	✓
	Community creation	✓	✓		✓			✓	✓	✓
**Feasibility**										
	Self-ownership of content	✓	✓	✓		✓	✓	✓	✓	✓
	Information and communication technology platform supplied by third party	✓	✓	✓	✓	✓	✓	✓	✓	✓
	Helpdesk and implementation support services supplied internally	✓	✓	✓	✓	✓	✓	✓	✓	✓
	Limited internal marketing capabilities	✓	✓	✓		✓	✓	✓		✓
**Viability**										
	Variable price for different packages of services, for a fixed amount of time, for caregivers, for health care organizations, and for municipalities and health insurers		✓						✓	
	Fixed price for access to information and services	✓		✓		✓	✓			
	Increasing the attractiveness of a larger health care platform				✓			✓		
	Supplying information and services at no cost							✓		✓

**^a^**Characteristic present in the case study.

##### Desirability

The interventions included as case studies were targeted at informal caregivers of people with dementia. However, many case studies have also decided to target formal care by developing aspects of their platforms for health care professionals:

Well you notice that there is a lot of demand from health care professionals. So people say “Gosh, we find this interesting” and recommend it to their clients, to know what is going on and what they are getting, before they give advice about which modules exist. It should be used that way. That the caregiver can initiate, but also the health care professional, and they both have need of that knowledge.

Respondent case study 5

Interestingly, even in blended interventions where the guidance of a health care professional is necessary, the eHealth platform was not targeted at these care professionals. In all cases, there remained a primary focus on the needs of informal caregivers. Next, a common characteristic was the overall relatively low pricing of eHealth (Table 2). The eHealth platform was often also incorporated in larger systems. These could be web-based platforms, such as Carenzorgt (Nedap) and case study 6 (redacted), or offline systems, such as DementieNL and Myinlife (Alzheimer Netherlands) and STAR eLearning (Dementia Meeting Centres):

I think an advantage of our situation at the university is that we have a national network of meeting centres. There are already 160 of them in the Netherlands, which come together once a year, where we inform them about all kinds of interesting developments for them, also the STAR course.

Respondent STAR

This integration significantly increased the visibility of the case study and provided a supportive structure. All case studies mentioned the time and effort needed to keep eHealth content relevant and up-to-date as a significant but necessary drain on resources. Finally, many case studies have emphasized the importance of creating a community around the eHealth intervention. These communities were places where caregivers could contact each other and share experiences about the eHealth intervention and dementia caregiving in general. These communities sometimes took place on a designated forum on the intervention website or via social media channels, such as a closed Facebook group.

##### Feasibility

Most of the cases were developed by groups with specific expertise in dementia and caregiving. Most of the studied cases chose to develop the content themselves (and hence owned this content) while hiring an external party to help build and maintain the web-based platform. Often, it was the expertise party who ran a helpdesk and support service for users, forwarding technical questions to the hired software party. In this case, these support tasks were either part of a research position at a university or on a volunteer basis. Most of the studied cases were limited in terms of marketing. In general, their aim seemed to be to sustain eHealth rather than scale it up:

So when regions report that this sounds good, they also want this, then we start. But we do not actively search for regions where we can implement OZOverbindzorg. We do not think that is societally appropriate. So no, they come to ask us.

Respondent OZOverbindzorg

This is because none of the cases aimed to make a profit. Payment was mentioned as a barrier multiple times, with respondents warning that setting up a secure payment system takes considerable time and effort and that their older demographic is not always comfortable with solutions such as PayPal.

##### Viability

In total, 4 different types of viability strategies were observed in 9 cases. Most interventions (Partner in Balance, STAR eLearning, OZOverbindzorg, and case study 6) opted to vary their prices for different *subscription packages* of services. They did this in terms of both content and volume. Other case studies (such as Thinkability) offered their eHealth interventions on the web for a fixed price, after which the buyer had access to the service indefinitely. Indeed, in this sample, Thinkability is the only intervention whose cost structure was centered around direct download from the internet by the caregiver, without mediation by an existing health care system. STAR eLearning and the case study 6 modules are also available for the caregiver to access without a health care organization, although these modules are at least partly integrated into existing dementia care systems, such as dementia meeting centers and a care organization, respectively. Finally, some cases, such as Carenzorgt and DementieNL (and Myinlife), made no revenue but instead increased the attractiveness of a larger health care platform or organization:

We mainly make software for health care institutions, especially in elderly care and disabled care and a bit in mental health care. Nine years ago, we thought it was important to involve the client’s family in the process. This also helps health care professionals do better. So we said, we will make this platform. We think it’s important for the entire infrastructure that we have this and offer it for free.

Respondent Carenzorgt

Finally, other case studies (such as DementieNL and Myinlife) existed to supply information and services at no cost to caregivers and were financed in large part through sponsorships.

#### Sustainability of Business Models

It was clear that although all the case studies were included because of their use in practice, the levels of use varied strongly. Table 4 lists the implementation phases and business models of the included case studies. The descriptions of these business models as well as the implementation phase of the intervention are based on the responses of the interview participants during the case study interviews. The descriptions also contain information on how the interventions are financed (in brackets).

**Table 4:** Implementation phase and business models of the case studies.

Implementation of case studies			Business models
**Sustainable implementation**			
	OZOverbindzorg	Subscription (paid by municipalities and health insurers)	
	Carenzorgt	Incentive (free to increase attractiveness of the larger platform)	
	STAR eLearning	Subscription (paid individually by the caregiver or in bulk by the organization, with varying options)	
	DementieNL (and Myinlife)	Sponsorship (free through sponsor support to Alzheimer Netherlands)	
**Developing implementation**			
	Partner in Balance	Grants, with a plan for subscription model (paid by municipalities and organizations)	
	Thinkability	Combination of fixed price and grants (one-time download from App Store and Google Play)	
	Case study 6	Combination of fixed price and grants (paid individually by the caregiver or in bulk by the organization, with varying options) for nonorganization members, free if organization member	
	Case study 5	Fixed cost (paid individually by the caregiver or in bulk by the organization, with varying options) for nonorganization members, free if organization member	
	Nachtrust bij Dementie	Grants (research project)	

### Lessons From Respondents

During the interviews, respondents from each case study were asked to formulate recommendations for future eHealth developers based on their experiences in bringing eHealth for dementia caregivers into practice. Textbox 1 lists the lessons learned, as reported by the case study interviews.

**Lessons learned by respondents.**

- Make use of an innovation consortium by involving health care providers, financers, users, and technical developers in every phase of development
- Provide a good fit with the intervention’s implementation context
- Ensure long-lasting marketing
- Start implementing and learning, rather than waiting and perfecting designs. In other words, start small and develop the interventions further based on user feedback
- Bigger implementation budgets are crucial
- Commercial collaboration is a big help (eg, marketing firms, information and communication technology companies, and sales and legal experts)
- Health insurer collaboration is beneficial to sustainable implementation through its potential to determine priorities and develop relevant and sustainable interventions
- Involve users in the whole process as much as possible, for example, through co-design and cocreation

## Discussion

### Principal Findings

This study identified a sample of 9 case studies on the implementation, use, and development of eHealth interventions for caregivers of people with dementia that are currently used in practice in the Netherlands. The main findings from interviews with representatives of these interventions showed that 4 cases were found to have developed sustainable business models (ie, business models that generate revenue for the upkeep and development of the intervention in the future). Overall, 5 cases were implemented in a more exploratory manner and relied on research grants to varying extents, although some had also developed preliminary business models. These findings have led to the following recommendations for the design, the implementing team, and the suggested implementation strategies of a core business model to help achieve the long-term implementation of eHealth interventions for caregivers of people with dementia.

### Design of eHealth Business Models

First, the design of the proposed core business model is based on the desirability, feasibility, and viability characteristics of the case studies. The first key element of this design concerns its desirability, specifically by incorporating eHealth interventions into larger, pre-existing health care contexts. The case study interview respondents mentioned that the fit of the interventions with existing organizational goals and contexts was an important part of intervention desirability, separately from any financial considerations. This is in line with previous studies on the implementation of eHealth interventions in other populations [35-37]. Indeed, contextual integration is an important part of many established implementation frameworks, including the Consolidated Framework for Implementation Research [38], Normalization Process Theory [39], and Open Innovation Theory [40].

The second key element of the proposed design concerns the similarities in the feasibility characteristics of the interventions. The case studies tend to supply the dementia-specific content and helpdesk of the eHealth internally, whereas ICT and software services are outsourced to an external party that does not own the content. In other words, the execution of these ICT-related key activities is shifted to key partners. Previous research has discussed the frequent outsourcing of services in ICT [41] and emphasized the importance of trust between involved parties in constructing these types of business models in eHealth [42]. These studies recommend knowledge sharing experiences between the involved parties to foster trust, which has been shown to help attain the benefits of outsourcing.

Finally, the third key element of the proposed core model’s design pertains to its viability. A prevalent, sustainable cost structure is a fixed price for access to the intervention’s information or services, which is paid by health care organizations. Another viable option for sustainability is integration into a larger platform that sponsors the eHealth intervention, so no cost is charged from the caregiver. Again, it is clear that when considering the viability of these business models, viewing the intervention within its health care context can facilitate its sustainable implementation [28]. This pertains to both the desirability of the intervention through its contextual fit and its financial viability through its use of revenue structures already in place. A good example of the successful application of these 3 core model elements is OZOverbindzorg, which makes use of fixed price, low cost, and equal buy-ins from collaborations between health insurers and municipalities per participating region. This is in line with the principles discussed in previous research, namely, that costs and benefits should be balanced between parties in eHealth, and one party should not disproportionally benefit from something that is financed by the other party [43]. Here, the benefits are shared between the care organizations and community well-being; therefore, it is fair that both equally contribute to the costs.

### Implementing Team

Next, the main finding of this study concerns the question of who is implementing these interventions. In this study, case studies that did not originate in an academic research context more successfully achieved sustainable implementation, compared with the case studies that originated in an academic research context. There is a noticeable absence of eHealth interventions that originated as research projects in the identified, sustainable financing models used in practice. Indeed, most academic interventions are constrained by expiring funding [44], and integration within existing structures is crucial. In this regard, this research underscores the need for more implementation funding and research into the business modeling of evidence-based eHealth interventions [45,46].

In addition, more traditional research methods such as randomized controlled trials are not time efficient or resource efficient and can impede researchers from reaching this implementation stage [47]. The use of alternative, more flexible research designs, with faster iteration and earlier consideration of implementation determinants, could also help overcome this barrier [48]. The benefits of sustainably implementing these academically developed eHealth interventions for caregivers of people with dementia include avoiding squandering public money on failed implementations, better allocation of research resources, and realizing the anticipated benefits for intended users [49].

### eHealth Implementation Strategy

Finally, an important question is, “Which strategies can help bring the proposed core business model into practice, and keep it in practice?” Here, it may be useful to gain inspiration from business. For instance, digital transformation strategies are a key part of bringing business models into practice by coordinating and prioritizing the many different aspects of digital transformation [50]. Previous research on digital transformation strategies has pointed to the importance of community creation in generating revenue through *freemium* business models [51]. A freemium business model involves “offering a basic version of the product or service free of charge, while the premium version is made available against additional payment” [52]. Community creation refers to forming and maintaining a community of intervention users who are in contact with each other through the intervention.

**Figure 3:**
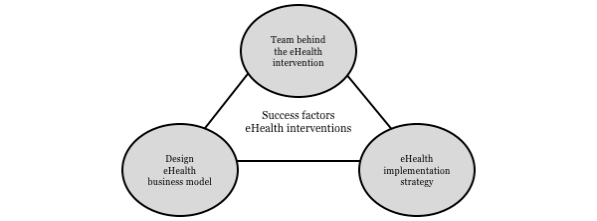
Success factors of the eHealth interventions for caregivers of people with dementia.

Although there was no clear example of a *freemium* business model in this sample, community creation was an important part of many of the included case studies, even more so among those grouped in the sustainable implementation category. Future research could test the proposed core model for external validity and investigate the effectiveness of community creation as part of a digital implementation strategy to increase the sustainability of eHealth interventions for caregivers of people with dementia. Here too, alternative, flexible research designs offer possibilities for comparing and evaluating innovative implementation strategies [53]. Figure 3 depicts the proposed core business model.

### Strengths and Limitations

This study helps alleviate a significant problem in the field of eHealth interventions for caregivers of people with dementia: a lack of information on financially viable, long-term implementation trajectories. By taking an intersectoral perspective and learning from interventions already being used in practice, this research is broader than most studies, which are often limited to studying interventions developed in an academic research context. This approach made use of a systematic method to select interventions for case studies based on expert opinion and predefined criteria. Finally, it is important to note the timeliness of this study. It is fair to say that in the context of the COVID-19 pandemic, there is a greater need than ever to provide caregivers of people with dementia with good web-based support options. The findings of this study provide concrete implementation lessons to aid eHealth developers who wish to implement and scale up eHealth interventions to support caregivers of people with dementia at a distance.

This study has several limitations. First, there is a possibility of selection bias, as the definition of this sample required responses from the authors’ own dementia and eHealth networks. As a result, the first possible bias is toward experts from academia, who are overrepresented in this sample. Second, there is likely a higher than average degree of sample familiarity with the authors’ own interventions, Partner in Balance and Myinlife, as the contacted experts belong to the same networks (such as INTERDEM) and are often exposed to other members’ research. Moreover, the authors acknowledge the potential bias in reporting the results concerning Myinlife and Partner in Balance, as they were involved in their development and evaluation. Third, it is likely that more interventions originating from the Southern Netherlands were included (as this is where the authors’ research group is based), whereas interventions from elsewhere in the Netherlands remained underrepresented. A second limitation concerns the potential self-report bias of the included case study respondents to emphasize positive aspects of their own interventions and minimize difficulties in the responses about their learned lessons. A final limitation concerns the fact that this study did not guarantee respondents’ total anonymity; because interventions would be referred to by name (unless respondents explicitly requested otherwise, as in case studies 5 and 8), the intervention could conceivably be linked to one of a few possible intervention respondents. This was discussed before the interview and agreed to by the respondents, who also provided a member check, approving the interview transcript. This lack of total anonymity could have impeded respondents from discussing more sensitive implementation topics candidly, out of fear of (social) repercussions from collaborators. Another possible consequence of this lack of anonymity is that the eHealth contact persons might not have wanted to share important details of their business plans to remain competitive.

### Conclusions

Case studies that did not originate from an academic research context seemed to achieve more sustainability, whereas case studies from academic research contexts experienced barriers to financial independence from research grants. Examining the common and differential characteristics of these case studies resulted in the proposal of a core business model for eHealth interventions for caregivers of people with dementia, derived from a sample of case studies currently being used in practice. This proposed core business model suggests increasing desirability, feasibility, and viability by integrating into larger structures; owning and supporting content internally while developing ICT services externally; and offering fixed, low-level pricing. Together with the origin of the case studies, these elements contributed to the sustainability of case studies. Finally, targeted digital transformation strategies, more intersectoral cooperation, and more financial incentives for research on sustainable business models are recommended to help future developers bring eHealth interventions for caregivers of people with dementia into practice.

## Appendix

Multimedia Appendix 1 Questionnaire.

## Abbreviations

ICT: information and communication technology
NVN: Nederlandse Vereniging voor Neuropsychologie (Dutch Association for Neuropsychology)
