# Supplementary material for: Business Models of eHealth Interventions to Support Informal Caregivers of People With Dementia in the Netherlands: Analysis of Case Studies
Source: JMIR Aging. 2021 Jun 3;4(2):e24724. doi: 10.2196/24724 (PMC8212631; doi:10.2196/24724)
Supplement: Multimedia Appendix 1 [file aging_v4i2e24724_app1.docx]

**Appendix. Semi-structured interview questions**

Desirability

1. Who is this intervention aimed at?
2. What is the advantage of this intervention for its clients?

Feasibility

1. What needs to be done (internal and external) to enable the intervention?

Viability

1. How does the intervention generate returns? License, advertisement, payment model…?
2. What are the major cost drivers and how are they linked to the revenue?

Other questions

1. How many people were involved?
2. Do you have any lessons about successful implementation that you want to share with us?
3. Have you gained inspiration from certain other interventions or theoretical frameworks?

Ending

1. Do you have any questions or things that would be interesting for me to know?
